# Impairments of Photoreceptor Outer Segments Renewal and Phototransduction Due to a Peripherin Rare Haplotype Variant: Insights from Molecular Modeling

**DOI:** 10.3390/ijms22073484

**Published:** 2021-03-27

**Authors:** Luigi Donato, Ebtesam Mohamed Abdalla, Concetta Scimone, Simona Alibrandi, Carmela Rinaldi, Karim Mahmoud Nabil, Rosalia D’Angelo, Antonina Sidoti

**Affiliations:** 1 Department of Biomedical and Dental Sciences and Morphofunctional Imaging, Division of Medical Biotechnologies and Preventive Medicine, University of Messina, 98122 Messina, Italy; ldonato@unime.it (L.D.); salibrandi@unime.it (S.A.); crinaldi@unime.it (C.R.); rdangelo@unime.it (R.D.); asidoti@unime.it (A.S.); 2 Department of Biomolecular Strategies, Genetics, Cutting-Edge Therapies, I.E.ME.S.T., 90139 Palermo, Italy; 3 Department of Human Genetics, Medical Research Institute, University of Alexandria, Alexandria 21526, Egypt; drebtesamabdalla@yahoo.com; 4 Department of Chemical, Biological, Pharmaceutical and Environmental Sciences, University of Messina, 98122 Messina, Italy; 5 Department of Ophthalmology, Faculty of Medicine, University of Alexandria, Alexandria 21526, Egypt; karim_nabil_ophth@yahoo.com

**Keywords:** Retinitis pigmentosa punctata albescens, *PRPH2*, rHTV, *RHO*, *RLBP1*

## Abstract

Background: Retinitis pigmentosa punctata albescens (RPA) is a particular form of retinitis pigmentosa characterized by childhood onset night blindness and areas of peripheral retinal atrophy. We investigated the genetic cause of RPA in a family consisting of two affected Egyptian brothers with healthy consanguineous parents. Methods: Mutational analysis of four RPA causative genes was realized by Sanger sequencing on both probands, and detected variants were subsequently genotyped in their parents. Afterwards, found variants were deeply, statistically, and in silico characterized to determine their possible effects and association with RPA. Results: Both brothers carry three missense *PRPH2* variants in a homozygous condition (c.910C > A, c.929G > A, and c.1013A > C) and two promoter variants in *RHO* (c.-26A > G) and *RLBP1* (c.-70G > A) genes, respectively. Haplotype analyses highlighted a *PRPH2* rare haplotype variant (GAG), determining a possible alteration of *PRPH2* binding with melanoregulin and other outer segment proteins, followed by photoreceptor outer segment instability. Furthermore, an altered balance of transcription factor binding sites, due to the presence of *RHO* and *RLBP1* promoter variants, might determine a comprehensive downregulation of both genes, possibly altering the *PRPH2* shared visual-related pathway. Conclusions: Despite several limitations, the study might be a relevant step towards detection of novel scenarios in RPA etiopathogenesis.

## 1. Introduction

Retinitis pigmentosa, also known as RP, refers to a group of inherited diseases characterized by retinal gradual atrophy, caused by photoreceptor cell death with possible involvement of adjacent cell layers, implying progressive deterioration of vision. The RP group includes more than 80 different forms of inherited eye disorders that can be classified on the basis of several criteria: age of onset, fundus appearance and other clinical features, causative gene, and inheritance pattern [1]. The age of onset is very variable and related to the specific form. Instrumental examination generally reveals widespread pigment deposits reaching the macular area, with thin vessels and a waxy pallor of the optic disc [2].

Such genetic disorders result from pathogenic variants in at least one of about 80 genes involved, and several non-coding RNAs were recently linked to RP etiopathogenesis [3,4]. RP can be inherited as an autosomal-recessive (about 50–60% of cases), autosomal-dominant (30–40%), or X-linked (5–15%) trait [5]. Additionally, very wide are the biochemical pathways involved in all different forms of RP, ranging from angiogenesis alterations [6] to mitochondria and ion channels impairments [7,8]. 

Retinitis punctata albescens (RPA) is one of the different forms of RP characterized by childhood onset night blindness, white retinal deposits, reduced visual acuity in the range 20/40, and areas of peripheral retinal atrophy (the macula is usually spared in early stages). In later stages, there may be atrophy of the retinal pigment epithelium, progressing to geographic atrophy of the macular pigment epithelium as the visual field becomes more constricted [9]. RPA is associated with variants in the *PRPH2* (OMIM gene: 179605; OMIM disease: 136880), *RHO* (OMIM gene: 180380; OMIM disease: 136880), *RLBP1* (OMIM gene: 180090; OMIM disease: 136880), and *RDH5* (OMIM gene: 601617; OMIM disease: 136880) genes [10,11]. In particular, the *PRPH2* gene encodes for the peripherin 2 that is a photoreceptor-specific transmembrane glycoprotein, a ~39 kDa member of the tetraspanin superfamily of proteins, necessary for the proper formation of both rod and cone photoreceptor outer segments (OSs) [12]. PRPH2 plays a role in the formation of the outer segment disc rim, and its loss leads to the absence of discs. Furthermore, PRPH2 has also been suggested to play a role in disc stability and disc shedding [13].

The aim of our study was to investigate the genetic cause of RPA in a family consisting of two affected Egyptian brothers, ages three and four, with the same typical phenotype and healthy consanguineous parents. After analysis of four known RPA causative genes, both brothers resulted in carrying three missense variants in *PRPH2* and two promoter variants in *RHO* and *RLBP1* genes, respectively. Found variants were genotyped in the probands’ parents, and then characterized first statistically and subsequently in silico to determine their possible consequences and association with diagnosed retinal pathology.

## 2. Results

### 2.1. Genotyping of Probands Revealed Variants in PRHP2, RHO, and RLBP1

We screened, first in the children and then in their parents, the four known genes associated with RPA, and found several variants. Three *PRPH2* missense SNPs were detected in a homozygous condition in both probands and in a heterozygous condition in both parents (rs390659: c.910 C > G, p.Gln304Glu and rs434102: c.1013 A > G, p.Asp338Gly), except one in the father that was in homozygosity (rs425876: c.929 G > A, p.Arg310Lys) (Figure S1). Additionally, one proband carried a 5′UTR variant, rs7984 (c.-26 A > G), in the *RHO* gene in a homozygous condition, and the same SNP was carried in heterozygosity in the second proband, as well as in the parents. Finally, both probands and the father carried another 5′UTR variant, rs3743384 (c.-70 G > A), this time in the *RLBP1* gene and in a heterozygous condition. The latter was absent in the mother.

### 2.2. Haplotype Analyses Shed Light on a PRPH2 Candidate, A Rare Haplotype Variant Associated to RPA

While *RHO* and *RLBP1* carried only one variant each, even if they were two SNPs with a not very high frequency in the Egyptian population (MAF *RHO* rs7984 = 0.057; MAF *RLBP1* rs3743384 = 0.906), *PRPH2* presented three variants, which alone should not be associated to RPA (MAF rs390659 = 0.297; MAF rs434102 = 0.296; MAF rs425876 = 0.020). Therefore, in order to hypothesize their effects as a rare haplotype variant (rHTV), a haplotype analysis was conducted. Comparisons of haplotype frequencies and associations between inferred haplotypes with RPA are summarized in Table 1.

The inferred haplotypes block by haplo.stats, consisting of the three analyzed *PRPH2* SNPs, resulted in significant differences between the case and controls, assuming recessive genetic traits (Bonferroni-adjusted *p*-value = 0.007). A significantly increased risk of RPA was observed with haplotype GAG, assuming recessive genetic traits (odds ratio = 55,771; 95% CI = 31,460–98,869; Bonferroni-adjusted *p*-value = 0.002). A borderline association, probably with protective effects, might be evidenced by CGA haplotype (Bonferroni-adjusted *p*-value = 0.052), the most frequent in controls (about 50%), but, as highlighted by the odds ratio = 1 and being not applicable relative CI, it needs an increased sample size to be correctly evaluated. The problem of controls’ over-representation probably determined the out-of-range OR in GAG haplotype association analysis by haplo.stats, so the Bayesian penalized regression of LBL permitted us to downplay such an error. In this way, logistic Bayesian LASSO confirmed an increased risk of RPA in subjects with the GAG haplotype (BF = 48.044; OR = 63.295; CI lambda = 0.5943–14.355; CI D = 0.9614–0.9997), even if the problem of small sample size, especially of cases, could not be removed.

### 2.3. Primary Structural and Biochemical Analyses Highlighted Possible Altered Chemical-Physical Features in Mutated PRPH2

The combined approach realized thanks to both CLC Workbench Main and Protean 3D software suggested an increase of mutated *PRPH2* molecular weight (39,271 Da versus 39,186 of wild-type protein). Moreover, the presence of selected variants determined a higher pI for 304Q and 310R, while a marked reduction was detected for 338D. Additionally, the net charge (pH = 7) of 304Q increased, while the same parameter for 338D strongly decreased. Another relevant chemical-physical feature that showed changed in the mutated protein regarded the local average hydropathy value, strongly reduced for both 310R and 338D. Such qualitative results were expanded by amphiphilicity (by Eisenberg) and hydropathy (by Kyte–Doolittle and Hopp–Woods) analyses that highlighted diffuse differences in the distribution of hydrophilic and hydrophobic residue groups in the mutated protein. Details of previously described results are available in Table 2.

### 2.4. Secondary Structure Analysis of Mutated PRPH2 Showed a Global Instable Protein

Although the secondary structures obtained using the Garneier–Robson, Chou–Fasman, and Deleage–Roux were not strongly consistent, several secondary structures (e.g., alpha helices, beta sheets, beta turns, coils, coiled coils, loops) resulted in a change comparing wild-type protein with the mutated one, especially in the aa range between 280–346 (Figure S2). Very interestingly, the flexibility analysis realized with the Karplus–Schulz approach showed the shifting of two flexible regions from aa 287–305 to 287–306, and from 309–316 to 310–316; these data were corroborated by the Median B-factor increase for all three considered SNPs (Table 3).

Furthermore, the presence of three found variants should shift the disordered regions upstream (analyzed with the JRONN algorithm), probably unable to form well-defined 3D structures, between 286–312 to 286–310 and between 328–346 to 327–346. Such a folding alteration prediction is supported by an increase of the stability–instability index between 288 and 319 aa, mainly determined by the G338D variant, as shown by its related change in the energy delta-E (DFIRE) value (1.664), with destabilizing effects. Finally, the surface probability (by Emini) analysis predicted the change in the surface exposition of several areas between aa positions 310 and 343. In detail, aa 310 became unexposed in mutated *PRPH2*, while 335 and 340 shifted to the surface. The only stable motif that remained unaffected in the presence of variants was the tetraspanin domain (aa 16–287).

### 2.5. Clash Analysis of the PRPH2 Mutated 3D Predicted Structure Evidenced a Possible Misfolding in Tetraspanin and MREG Interacting Domains

**Figure 1:**
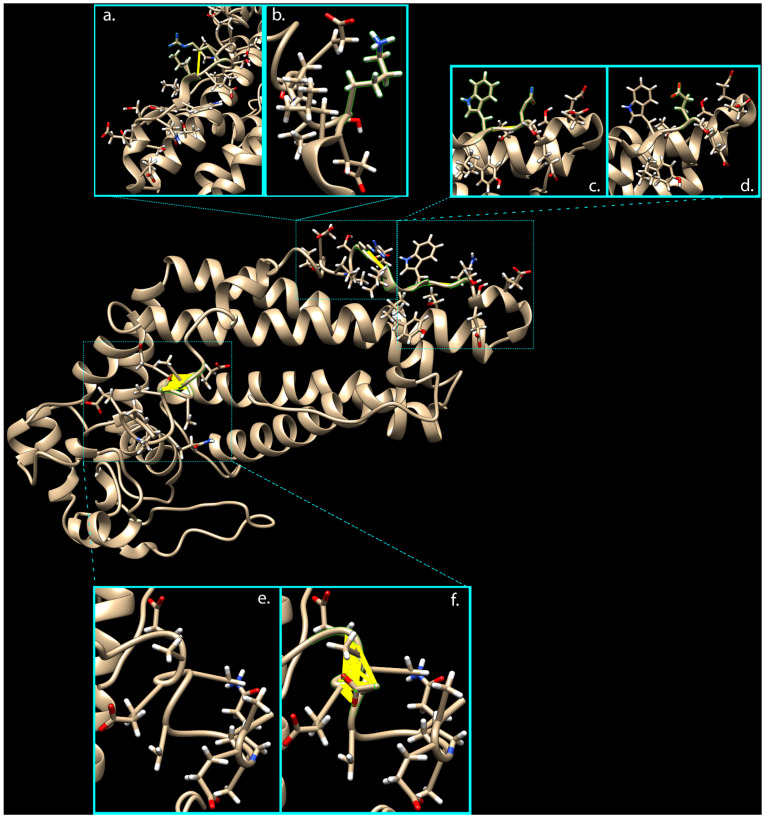
Clash analysis of the *PRPH2* mutated 3D predicted structure. Thanks to ChimeraX software, it was possible to predict presence of clashes in 3D protein structure due to the three variants carried by mutated *PRPH2*. Turquoise boxes show 3D protein domains implicated in clashes (yellow lines), considering wild-type (**b**,**d**,**e**) versus rs390659 (**a**), rs425876 (**c**), and rs434102 (**f**) involved areas, respectively.

Due to the unavailability of the *PRPH2* 3D structure in the PDB Molecule Database, PHYRE2, RaptorX, and i-TASSER servers were used to predict and model multiple domains of wild-type and mutated proteins with high confidence based on the high scoring template. Prediction by i-TASSER gave the most confident results, showing a normalized Z-score of 8.63 for the wild-type structure and a value of 8.45 for the mutated one. The comparative analysis of 3D models by ChimeraX software highlighted the presence of 32 interatomic clashes (unfavorable interactions where atoms are too close together; index of close contacts), involving aa in positions 306, 308, and 333–336, as well as in adjacent zone (areas with atoms/bonds presenting a distance of less than 4 Å from considered variants), consisting of aa in positions 285, 288, 298, 300–315, and 333–346 (Figure 1 and Table S1).

The introduction of these clashes in the mutated protein, mainly caused by the G338D variant, might misfold the final portion of the tetraspanin domain (this time with the prevalent effect of E304Q) and the whole MREG interacting domain (aa 341–346, from UniProt Database).

### 2.6. Variants in RHO and RLBP1 Promoters Might Alter Their Expression, Indirectly Involving PRPH2

The predictive analysis of proband’s *RHO* and *RLBP1* mutated promoters revealed the possible loss of one group of TF binding sites (NR2F2, RXRA, USF3, MYC, MAX, MLXIPL) for the first gene due to the presence of rs7984, while the substitution of the DEAF1 binding site with the SLC2A4RG one is attributable to rs3743384 for *RLBP1* (for further details, see Table 4).

These results suggest a probable transcription variation due to the altered balance of TF binding properties. Furthermore, it is important to understand the relationship between the analyzed TFs, and how each one could influence the others. Such an aspect was investigated by Cytoscape pathway analysis, along with its GENEMANIA plug-in, from which arose a strong network involving all TFs in exam (Figure S3). The relevance of just the described data could become really interesting if it considers that *PRPH2*, *RHO*, and *RLBP1* are co-expressed and co-localized in the same tissues, sharing several common pathways (Figure S3).

## 3. Discussion

Retinitis pigmentosa punctata albescens is a recessive autosomal disease characterized by a well-known phenotype consisting of white dots in the fundus, progressive macular atrophy, peripheral vision loss, and early nyctalopia. We studied an Egyptian family, made of consanguineous parents, whose two children suffered from night blindness from birth. As in our case, the diagnosis of RPA can be challenging in early childhood. Thus, a mutational screening is fundamental to find genetic reasons of disease etiopathogenesis. We identified three missense variants in the *PRPH2* gene coding sequence (rs390659, rs425876, and rs434102), and one in both promoters of *RHO* (rs7984) and *RLBP1* (rs3743384) genes.

*PRPH2*, also known as *RDS* (retinal degeneration slow), codifies for the peripherin-2 glycoprotein, which is essential in the morphogenesis of stacked bilaminar discs of the photoreceptor outer segment plasma membrane [14]. Each disc consists of a phototransductional structure, the lamella, and a rim that maintains the flattened morphology. Alteration of this protein results in cellular disorganization and cellular apoptosis activation, affecting vision [15]. More than 90 pathogenic variants in *PRPH2* are associated with retinal dystrophies, including retinitis pigmentosa punctate albescens [16]. The protein encoded by *RHO*, RHOdopsin, is a photopigment essential for vision in low-light conditions. RHOdopsin is involved in the initial steps of the visual transduction cascade; it binds to 11-cis retinal and is activated when light hits the retinal molecule [17]. Moreover, it is required for photoreceptor cell viability after birth [18], and is involved in cargo trafficking to the periciliary membrane super-pathway [19,20]. It represents more than 80% of the constituent protein of the rod outer segment membrane, and genetic variants are known to be causative of several forms of inherited dystrophies, such as RPA [21].

In humans, mutations in *RLBP1*, the gene encoding the cellular retinaldehyde-binding protein (CRALBP), represent the principal genetic causes of RPA [22]. CRALBP binds the vitamin A derivatives 11-cis retinol and 11-cis retinal, accelerating the rate of the aldehyde form isomerization to 11-cis retinol and acting as a key regulator of the visual cycle [23]. CRALBP is particularly abundant in the retinal pigment epithelium and the Muller glial cells, and its deficiency leads to considerably delayed dark adaptation [24].

Except for rs3743384 in one affected member, all of the other detected variants are carried in a homozygosity condition in both probands, strongly different from the parents. Moreover, all cited variants are associated to RPA phenotypes in several genetic databases, like Ensembl (http://www.ensembl.org, accessed on 23 March 2021) and ClinVar (https://www.ncbi.nlm.nih.gov/clinvar/, accessed on 23 March 2021), albeit without well-defined effects. Only one variant, the *PRPH2* rs434102, was found in the Human Gene Mutation Database (HGMD, http://www.hgmd.cf.ac.uk/ac/index.php, accessed on 23 March 2021), but, even if evaluated as causative of RPA in the first instance, it now appears to be retired.

Consequently, our hypothesis is based on the combined effects exerted by a possible haplotypic *PRPH2* block, the effects of which might be indirectly increased by potential regulative SNPs carried by *RHO* and *RLBP1*. It has been widely acknowledged that haplotype analysis in association studies can provide much more useful information than the information derived from single polymorphisms analysis [25]. The primary reason for considering the haplotype organization of variation resides in the point that the folding kinetics, stability, and other physical features of a protein may depend on interactions between pairs or higher-order combination of aminoacidic sites; if such interactions are relevant, then haplotypes are of direct biological importance. In this scenario, the strong linkage disequilibrium between rs390659 and rs434102 (r^2^ = 0.997 and D’ = 1.000, from Ensembl) permitted us to consider them as the driving tagSNP, homozygous for probands only, the consequences of which could be worsened by rs425876. Such a hypothesis was improved by haplotype analyses, which showed that the combination of all three analyzed *PRPH2* SNPs could form a rare haplotype variant (rHTV). It was recently known that rHTV made of common SNVs is more likely to tag rSNVs not genotyped in GWAS, providing greater power to detect rSNVs than standard collapsing methods [26]. Therefore, even in studies like ours, where haplotype association is not the goal per se, haplotypes provide a biologically sensible way to investigate the association with rSNVs to gain power. In order to correctly interpret the real meaning of rHTVs, they must not be considered as pooled into a single variant [27], as the latter can suffer from power loss, especially if the effects of the pooled rHTV might be conflicting (i.e., both risk and protective haplotypes exist) [28]. Recently, novel approaches considering both individual rHTVs and their directional effects have been suggested [29]. Among them, the haplo.stats EM and the more suitable LASSO approaches highlighted the possible association of GAG homozygous haplotype, present in both family probands, but absent in both parents and in most of the healthy population, with RPA. Such a hypothesis is supported by mutated *PRPH2* bioinformatic analysis, which highlighted a serious increase of structure instability in both secondary and tertiary levels that might lead to functional loss. All three variants reflect their effects on the C-terminal domain of *PRPH2*, the most divergent and intrinsically disordered region within the tetraspanin family of proteins, that may also confer functional specificity to these proteins [30]. Intrinsically disordered regions (IDRs) represent protein regions with high flexibility, and, even if they may lack a defined secondary or tertiary structure, they are still able to provide advantages in protein–protein interactions, including faster on and off rates of binding, a larger hydrodynamic radius, high binding specificity, and the ability to adopt different conformations depending on the binding partner, permitting the performance of several important biological functions [31]. Very interestingly, recent studies have revealed a high propensity for IDRs to undergo post-translational modification, particularly phosphorylation [32]. Thanks to disordered C-terminal, peripherin-2 can be transiently associated with numerous other proteins along the length of the OS, such as melanoregulin (MREG). It was experimentally established that MREG is essential for disk plasma membrane fusion transient inhibition, suggesting a regulative role for this protein in OS renewal processes. Additionally, peripherin-2/MREG complex links the disk rim to the cGMP-gated channel of the plasma membrane through glutamic acid-rich protein, GARP, thereby stabilizing the OS structure [33]. Therefore, the instability index reduction of the mutated *PRPH2* region involving aa 288–319 (88.72 versus 92.59 of wild-type protein), along with the destabilizing effect on protein–ligand complexes highlighted by DFIRE energy function and the increased number of clashes in 3D protein, allows us to hypothesize that the contemporary presence of three detected *PRPH2* variants might misfold the protein C-terminal, altering its binding with MREG and other outer segment proteins needed for disc vitality and consequent phototransductional ability.

A proposed idea might be corroborated by the possible effects of promoter variants in *RHO* (rs7984) and in *RLBP1* (rs3743384), consisting of the high probable loss of several activator (NR2F2 [34], USF3 [35,36], MYC [37,38,39,40], MLXIPL [41], and DEAF1 [42]) and repressor (RXR-alpha [43], MAX [44]) binding sites, with the creation of one only binding site for the repressor SLC2A4RG [45]. Such data, completed by Cytoscape analysis, permitted us to propose a hypothesis based on a probable global downregulation of both genes, as a result from an altered balance of all considered TFs, most of which could present a mutual influence in determining the final effect. Therefore, the final consequences of the *RHO* and *RLBP1* possible downregulation could imply a reduced or defective binding of *RHO* to *PRPH2* [46], with the additional effect of a possible insufficient oxidation of 11-cis-retinol, impairing the phototransduction cascade.

## 4. Methods

### 4.1. Clinical Data

The probands, two Egyptian male children aged three and four, sons of a consanguineous Egyptian couple, were presented to the ophthalmological clinic at the Human Genetics Department, Medical Research Institute of Alexandria University, with a diagnosis of retinitis punctata albescens. Nystagmus was observed in both children soon after birth. Fundus examination of both kids revealed normal anterior segment bilaterally and insignificant astigmatic refractive error, while the posterior segment already showed a bilateral pale disc with diffuse retinal whitish pigmentation (Figure S1). Flash visual evoked potential (VEP) analysis, realized twice for each eye separately according to the ISCEV standards (latest update) at a flash frequency of 7.3 Hz, showed no consistent or reproducible response for both eyes, evidencing an affection of the visual pathway on stimulating each eye. Furthermore, a flash electroretinogram (ERG), carried out for both eyes under photopic conditions and after 20 min of dark adaptation, showed a and b waves under both photopic and scotopic conditions, followed by small amplitude responses, evidencing an affection of rod- and cone-mediated function on both sides (Figure S4).

The patients’ family, consisting of father and mother, was evaluated by the same clinical and instrumental analyses, and resulted in being healthy. Both parents did not manifest slow dark adaptation, and showed a visual acuity of 20/20, a normal visual field, and a clean fundus. The family pedigree is shown in Figure S5.

We have screened all four known retinitis pigmentosa punctate albescens causative genes (*PRPH2*, *RHO, RLBP1,* and *RDH5*), and we have found no associated or causative variants (HGMD Professional was the most important and updated database we considered), except those candidates that we analyzed in this paper. 

### 4.2. Mutational Analysis

DNA was extracted from peripheral blood by using a QIAamp DNA Blood Midi Kit (Qiagen, Hilden, Germany) according to the manufacturer’s protocol. *RHO* (five exons), *RDH5* (five exons), *PRPH2* (three exons), and *RLBP1* (nine exons) genes were amplified using primers designed according to the genes’ published nucleotide sequence of GenBank (accessed on 22 March 2021). Sequences of primers are available upon request.

PCR amplifications were carried out in a 50 µl solution containing 2 µl of each primer (10 µM), 0.8 µg of genomic, and 1.5 U MyTaq DNA Polymerase (Bioline, London, UK). After an initial denaturation step at 95 °C for 1 min, the samples were subjected to 35 cycles of amplification consisting of 15 s of denaturation at 95 °C and 10 s of annealing. The annealing temperature was optimized for each primer set. Following PCR, 5 µl of amplified product was examined by electrophoresis on a 1% agarose gel. Direct sequencing was then performed using BigDye Terminator v3.1 chemistry on a 3500 Genetic Analyzer (Thermo Fisher Scientific, Waltham, MA, USA). Molecular screening of all genes was performed in the probands; then, detected variants were also searched in the other family members.

### 4.3. Haplotype Statistical Analysis

Potential disease-associated variants were confirmed by evaluating the frequency distribution of the Egyptian population, using several databases (ExAC [47], Ensembl [48]), and assessing 200 control samples that have the same ethnic origins as the analyzed family.

Afterwards, inferred haplotype block construction based on the three *PRPH2* SNPs was realized using the JAVA open source software Haploview [49], applying the Gabriel confidence interval method option. Then, two different statistical approaches were exploited to determine haplotype frequencies and to conduct joint effect modeling within each haplotype block, finally testing for association with RPA. The first was based on the R package called haplo.stats, which used an expectation maximization algorithm to establish which haplotypes showed significant differences between cases and controls, using a haplotype-specific score [50]. This score calculated a statistic that can be tested for significance using a chi-squared distribution with degrees of freedom equal to the number of inferred haplotypes existing in each haplotype block. Soon after, association with RPA was tested by applying the regression-based method for binary (case control) responses [51]. The second approach used another R package called LBL, based on logistic Bayesian LASSO methodology [52], including the likelihood, priors, and estimation of posterior distributions using Markov Chain Monte Carlo (MCMC) methods and an association test procedure. This Bayesian version of penalized regression is able to minimize the effects of unassociated (especially common) haplotypes to obtain enough noise reduction and more easily detect the signals contained in the associated rare haplotypes. In both approaches, for each haplotype block, the joint effects model was fitted, in which each haplotype was compared to the most frequent haplotype (used as a reference). Previous analyses were conducted with the assumption of recessive genetic traits, and included haplotypes with a frequency greater than 1%. The Bonferroni correction was, finally, applied to account for multiple testing.

### 4.4. In Silico Analyses

In order to investigate the possible effects of found variants on the coding sequence and their impact on *PRPH2* protein, CLC Main Workbench 21.0.3 software [53] was exploited. Then, the comparative secondary structure prediction of the wild-type and mutated protein sequence was realized by Protean 3D software [54], also evaluating hydropathy, stability, transmembrane properties, cleavage sites for proteases and chemicals, and functional domains scanning Prosite and Interpro databases. The prediction of the tertiary structure has been realized thanks to the Phyre2 [55], Raptor X [56], and Iterative Threading ASSEmbly Refinement (i-TASSER) hierarchical approach [57]; 3D structures of wild-type and mutated *PRPH2* were then analyzed by UCSF ChimeraX [58] to visualize conformational changes and variations in intramolecular contacts. Afterwards, in order to highlight the mutual involvement of *PRPH2*, *RHO,* and *RLBP1* in the same eye-related pathways, a deep analysis with Cytoscape [59] and its plug-in Genemania [60] was performed. Finally, 5′-UTR found variants were analyzed for their possible impact on *RHO* and *RLBP1* expression by GeneXplain [61] and Genomatix (trial version, https://www.genomatix.de/, accessed on 23 March 2021) web platforms.

## 5. Conclusions

Our results suggest a probable misfolding of *PRPH2* protein in probands due to the contemporary presence of the three missense detected variants, acting as a pathogenic rHTV. Particularly, the C-terminal appears to be the most impaired, determining a possible alteration of *PRPH2* binding with melanoregulin and other outer segment proteins. Compromising of such a function could lead to instability of the OS structure, especially arresting or impairing its renewal processes, resulting in RPA typical retinal degeneration. Moreover, we speculated that an altered balance of TF binding sites, due to the presence of c.-26 A > G and c.-70 G > A in *RHO* and *RLBP1* promoters, respectively, along with the possible interaction of involved transcription factors, could determine a comprehensive prevalence of repressive activity, rather than enhancing activity, resulting in a downregulation of both genes. Such down-expressions might be reflected in the possible physical interaction between both gene-encoded products and *PRPH2*, or, more plausibly, altering a shared visual-related pathway.

Even if the in silico analyses predicted the misfolding of peripherin-2 and a possible expression reduction of *RHO* and *RLBP1* promoters due to the effects exerted by all analyzed variants, we cannot assert with certainty that the same effect, in vitro and in vivo, is limited to their presence. For example, we cannot be sure of *PRPH2* binding property alterations, nor exclude the involvement of other factors into the altered expression of *RHO* and *RLBP1*; we also cannot ignore the possible phenotypic distinct pathological traits as the effects of never-associated mutated genes. Therefore, further experiments (e.g., dual-luciferase reporter assay [62] and ChIP-Sequencing of involved TFs, NanoBiT™ Complementation Assay) will be needed to confirm the effects of *PRPH2*-detected variants on protein folding and binding activity. For example, testing the expression of the mutant *PRHP2* carrying the three analyzed variants in some cultured non-neuronal cell lines (e.g., HEK293) could help to determine whether these mutants can cause cell trafficking dysfunction, misfolding, or changed cellular localization. Furthermore, the role of single transcription factors and reciprocal interactions probably involved in *RHO* and *RLBP1* down-regulation have to be tested. Likewise, the influence of *RHO* and *RLBP1* on *PRPH2* functions or shared pathways might be clarified. Moreover, another limitation of our study is related to the statistical power, giving rise to the need to replicate our findings with a larger sample size in order to ensure the effectiveness of our results. Despite this, considering the possible RPA-associated effects exerted by an rHTV, together with modifier gene expression alterations, might be a relevant step towards the detection of novel scenarios in RPA etiopathogenesis.

## Figures and Tables

**Table 1:** Comparisons of haplotype frequencies and associations between inferred haplotypes with RPA. Haplotype analysis by haplo.stats and LBL models highlighted a possible association of GAG rHTV with RPA. Haplo.stats values legend: Haplotypes # = the first column containing the haplotypes used in the analysis. Hap-Score = score statistic for the association of haplotype with the binary trait; adj. *p*-val (Bonferroni) = *p*-value for the haplotype score statistic, adjusted after Bonferroni correction; pool.hf = estimated haplotype frequency for cases and controls pooled together; control.hf = estimated haplotype frequency for control group subjects; case.hf = estimated haplotype frequency for case group subjects; glm.eff = the haplo.glm function modeled the haplotype effects as a baseline (Base) or additive haplotype effect (Eff); OR.lower = lower limit of the odds ratio confidence interval; OR = odds ratio based on the haplo.glm model estimated coefficient for the haplotype; OR.upper = upper limit of the odds ratio confidence interval. LBL values legend: BF = vector of Bayes factors for all regression coefficients; if BF exceeds a certain threshold (e.g., 2 or 3), association may be concluded. OR = vector of estimated odds ratios of the corresponding haplotype against the reference haplotype; this is the exponential of the posterior means of the regression coefficients. CI.OR (lower and upper) = 95% credible sets for the ORs. If CI.OR excludes 1, association may be concluded. CI.lambda (upper and lower) = 95% credible sets for the lambda parameter, that is the penalty coefficient. CI.D (upper and lower) = 95% credible sets for the D parameter, which is the within-population inbreeding coefficient.

GLOBAL SCORE STATISTICS												
global-stat = 7.7253		df = 2		adj. *p*-val (Bonferroni) = 0.007004		N° Controls = 152		N° Cases = 2				
Haplotype, scores, *p*-values, hap-frequencies (hf), and odds ratios (95%CI) --- haplo.stats												
Haplotype #	rs390659	rs425876	rs434102	Hap-Score	adj. *p*-val (Bonferroni)	pool.hf	control.hf	case.hf	glm.eff	OR lower	OR	OR upper
2	C	G	A	−1.40512	0.0533	0.49351	0.50000	NA	Base	NA	1	NA
1	C	A	A	−0.92901	0.1176	0.29870	0.30263	NA	Eff	1	1	1
3	G	A	G	2.77945	0.0018	0.20779	0.19737	1	Eff	31460	55771	98869
Haplotype, Bayesian factors, and odds ratios (95%CI) --- LBL												
Haplotype #	rs390659	rs425876	rs434102	BF	OR	CI.OR lower	CI.OR upper	CI.lambda lower	CI.lambda upper	CI.D lower		CI.D upper
2	C	G	A	/	/	/	/	0.5862	1.4365	0.9604		0.9997
1	C	A	A	0.9626	0.5904	0.0213	5.3881					
3	G	A	G	5.0203	6.6699	1.0921	214.0648					

**Table 2:** Structural features changing between wild-type and mutated *PRPH2*. Protean 3D and CLC Main Workbench results evidenced several structural changes in wild-type versus mutated *PRPH2* protein, with details of involved aa.

Structural Feature	Wild-Type Protein	Mutated Protein
Weight (da)	39,186	39,271
Absorption at 280nm 0.1% (= 1 g/L)		
Non-reduced cysteines	1891	1895
Reduced cysteines	1873	1877
Count of hydrophobic (A, F, G, I, L, M, P, V, W) residues	175	174
Count of hydrophilic (C, N, Q, S, T, Y) residues	95	96
Pfam domain result	Tetraspanin (aa. 16–287)	
Uniprot domain result	Interaction domain with Melanoregulin (MREG) (aa. 341–346)	
Instability index	92, 59	88, 72
Amphiphilicity and hydropathy	Changes in aa. 26–28, 106, 181–184, 209, 258–261, 272, 307–315, 336	
Flexibility	287–305, 309–316	287–306, 310–316
Disorder	286–312, 328–346	286–310, 327–346
Surface probability	Three residues (aa. 310–312)	Two residues (aa. 311–312)
	/	One residue (aa. 335)
	Three residues (aa. 341–343)	Four residues (aa. 340–343)

**Table 3:** Biochemical and physical change prediction between wild-type and mutated *PRPH2*. Analyses of biochemical and physical parameters by Protean 3D showed an increased global instability from wild-type to mutated *PRPH2* protein. Bold text highlights parameters with differences.

Feature	Wild-Type	Rs390659	Wild-Type	Rs425876	Wild-Type	Rs434102
AA change	E304Q—GLUTAMINE (GLN)	GLUTAMIC ACID (GLU)	K310R—ARGININE (ARG)	LYSINE (LYS)	G338D—ASPARTIC ACID (ASP)	GLYCINE (GLY)
Position	304	304	310	310	338	338
Type	L-PEPTIDE LINKING	L-PEPTIDE LINKING	L-PEPTIDE LINKING	L-PEPTIDE LINKING	PEPTIDE LINKING	**L-PEPTIDE LINKING**
MW [g/mol]	147.129	**146.144**	147.195	**175.209**	75.067	**133.103**
Net charge [pH = 7]	−1	**−0.01**	0.99	0.99	0	**−1**
pI	4	**5.52**	8.75	**9.75**	5.52	**3.8**
Average hydropathy	−3.5	−3.5	−3.9	**−4.5**	−0.4	**−3.5**
Aliphatic index	0	0	0	0	0	0
A₂₈₀ (ox.)	0	0	0	0	0	0
A₂₈₀ (red.)	0	0	0	0	0	0
ε₂₈₀ [M⁻^1^ cm⁻^1^]	0	0	0	0	0	0
φ	−62.5°	−62.5°	−66.3°	−66.3°	−162.2°	−162.2°
ψ	15.4°	15.4°	32.9°	32.9°	−2.7°	−2.7°
ω	111.0°	111.0°	123.8°	123.8°	161.5°	161.5°
Median B-factor	10.18	**25**	12.28	**25**	10.9	**17.95**
Effect on protein structure		**stabilizing**		**stabilizing**		**destabilizing**
Delta_energy		**−0.554**		**−0.143**		**1664**
Potential		DFIRE-A		DFIRE-A		DFIRE-A

**Table 4:** Genomatix analysis revealed TF binding sites’ loss/gain determined by examined variants. Predictive analysis of proband’s *RHO* and *RLBP1* mutated promoters revealed the possible loss of one group of TF binding sites (NR2F2, RXRA, USF3, MYC, MAX, MLXIPL) for the first gene due to the presence of rs7984, while the substitution of the DEAF1 binding site with the SLC2A4RG one is attributable to rs3743384 for *RLBP1*. Opt. thresh. = the optimized value defined in a way that a minimum number of matches is found in non-regulatory test sequences (i.e., with this matrix similarity, the number of false positive matches is minimized). Core sim. = the core sequence of a matrix is defined as the (usually four) consecutive highest conserved positions of the matrix. The core similarity is calculated basing on the core sequence. Matrix sim. = the matrix similarity is calculated only if the core similarity reaches a user-defined threshold (core similarity).

Gene	SNP ID	Lost/ New	Family/Matrix	TF Name	Family Info	Further Info	Opt. thresh.	Start pos.	End pos.	Strand	Core sim.	Matrix sim.
***RHO***	rs7984	lost	V$NR2F/COUPTFII.01	NR2F2	Nuclear receptor subfamily 2 factors	Chicken ovalbumin upstream promoter transcription factor 2, NR2F2 homodimer, DR1 sites	0.8	490	514	+	1	0.828
		lost	V$RXRF/RXRA.01	RXRA	RXR heterodimer binding sites	Retinoid X receptor alpha homodimer, DR1 sites	0.83	492	516	+	1	0.894
		lost	V$EBOX/USF.03	USF3	E-box binding factors	Upstream stimulating factor	0.89	494	510	-	1	0.904
		lost	V$EBOX/MYCMAX.03	MYC and MAX	E-box binding factors	MYC-MAX binding sites	0.91	495	511	+	0.842	0.919
		lost	V$CHRE/CHREBP_MLX.01	MLXIPL	Carbohydrate response elements, consist of two E box motifs separated by 5 bp	Carbohydrate response element binding protein (CHREBP) and Max-like protein X (Mlx) bind as heterodimers to glucose-responsive promoters	0.82	500	516	+	1	0.883
***RLBP1***	rs3743384	lost	V$DEAF/NUDR.01	DEAF1	Homolog to deformed epidermal autoregulatory factor-1 from D. melanogaster	NUDR (nuclear DEAF-1 related transcriptional regulator protein)	0.75	186	204	-	1	0.801
		new	V$HDBP/HDBP1_2.01	SLC2A4RG	Huntington’s disease gene regulatory region binding proteins	Huntington’s disease gene regulatory region-binding protein 1 and 2 (SLC2A4 regulator and papillomavirus binding factor)	0.84	189	207	-	1	0.863

## Data Availability

All data generated or analyzed during this study are included in this published article and its supplementary information files.

## Supplementary Materials

Supplementary Materials can be found at https://www.mdpi.com/article/10.3390/ijms22073484/s1. Figure S1. Probands’ ophthalmological exams; Figure S2. Egyptian family pedigree; Figure S3. Partial DNA sequence analysis of *PRPH2*, *RHO,* and *RLBP1* genes; Figure S4. Secondary structure and biochemical comparisons between wild-type and mutated *PRPH2* by Protean software; Figure S5. Cytoscape pathway analyses of three analyzed genes and transcription factors with probably altered binding sites; Table S1. Clash analysis of *PRPH2* mutated 3D predicted structure.
