# Supplementary material for: Impairments of Photoreceptor Outer Segments Renewal and Phototransduction Due to a Peripherin Rare Haplotype Variant: Insights from Molecular Modeling
Source: Int J Mol Sci. 2021 Mar 27;22(7):3484. doi: 10.3390/ijms22073484 (PMC8036374; doi:10.3390/ijms22073484)
Supplement: Supplementary file 1 [file ijms-22-03484-s001.pdf]

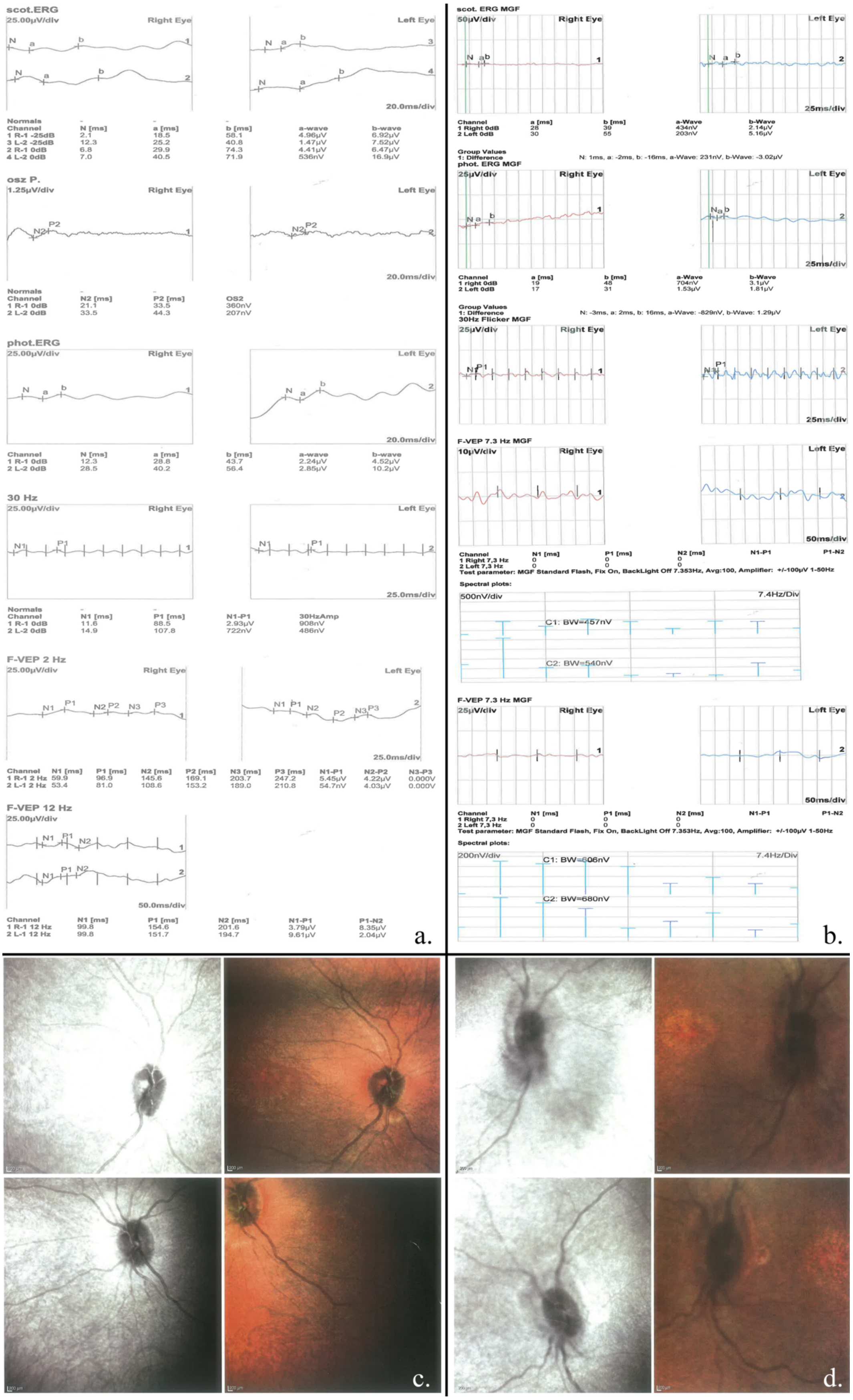

Figure S1. Probands' ophthalmological exams. ERG of patient III4 showed bilateral moderate to marked subnormal amplitude with latency of photopic (cone) with poor flicker 30HZ response, bilateral marked subnormal scotopic and maximal combined response with formed oscillatory potentials, and bilateral moderate to marked latency with subnormal amplitude of VEP response (a). ERG of patient III5 showed present a and b waves, under both photopic and scotopic conditions, followed by small amplitude responses, while VEP analysis showed no consistent or reproducible response for both eyes (b). Fundus analysis of both kids (III4 box c, III5 box d) revealed normal anterior segment bilaterally, insignificant astigmatic refractive error, while the posterior segment already showed bilateral pale disc with diffuse retinal whitish pigmentation.

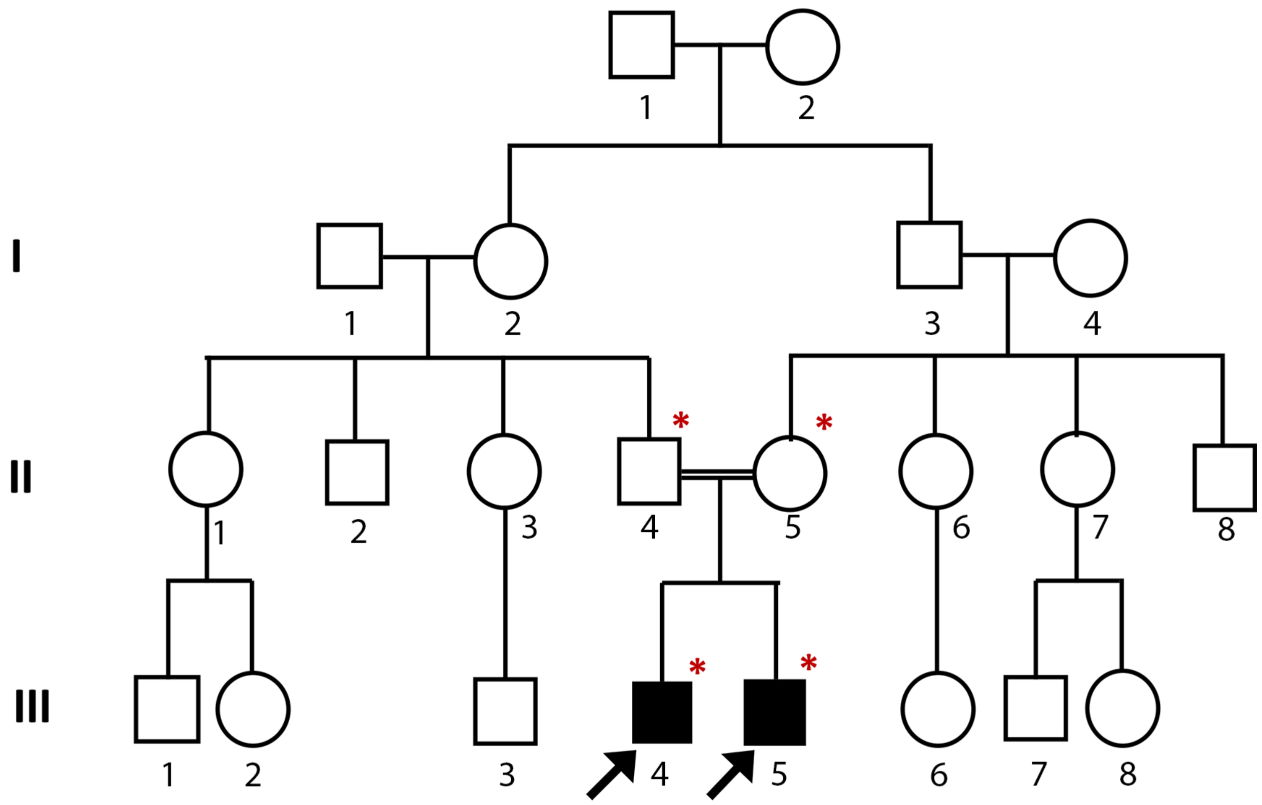

Figure S2. Egyptian family pedigree. A multi-generation Egyptian family in which RPA is segregating as an autosomal recessive trait. Two affected kids (III4 and III5) and two consanguineous carrier parents (II4 and II5) were screened for known causative genes mutations.

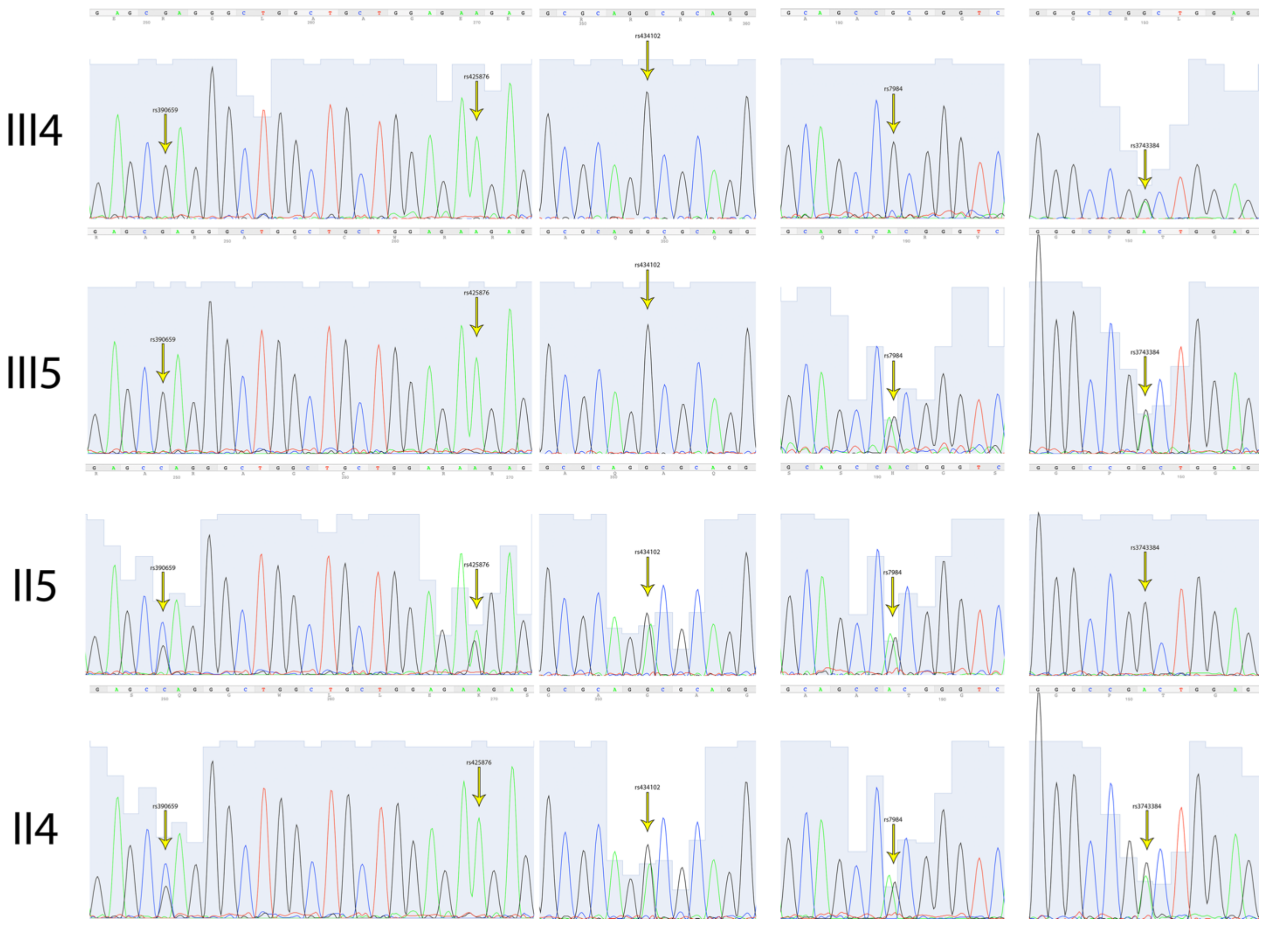

Figure S3. Partial DNA sequence analysis of *PRPH2*, *RHO* and *RLBP1* genes. Sanger sequencing of PRPH2 revealed the presence of c.910 C>G (rs390659) and c.1013 A>G (rs434102) in heterozygous condition in both parents (II4 and II5), while c.929 G>A (rs425876) is in heterozygosity in mother (II5) and in homozygous condition in father (II4). Furthermore, one proband (III4) carries the RHO c.-26 A>G 5′UTR variant (rs7984) in homozygous condition, and the same SNP is carried in heterozygosity in the second proband (III5), as well as in parents (II4 and II5). Finally, both probands (III4 and III5) and father (II4) carry the RLBP1 c.-70 G>A 5′UTR variant (rs3743384) in heterozygous condition. The latter variant is, instead, absent in mother. Variants are highlighted by arrows.

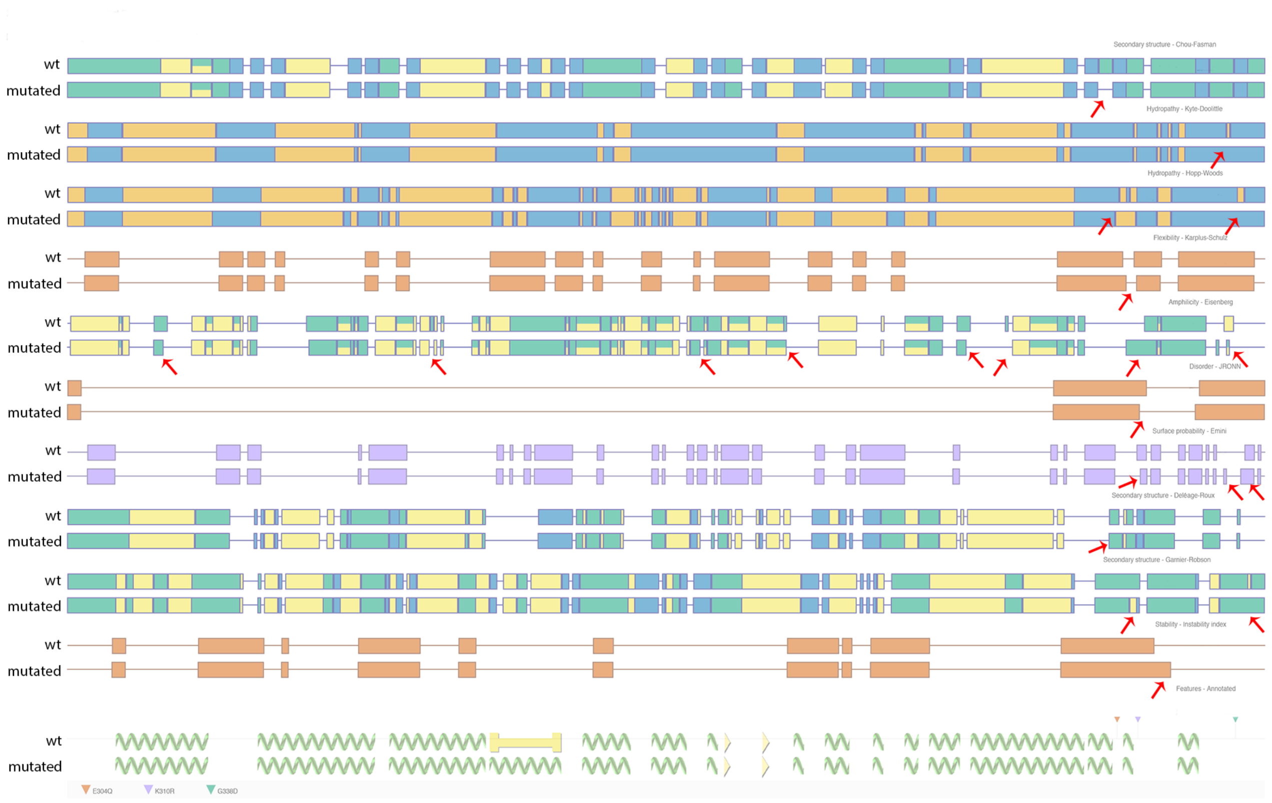

Figure S4. Secondary structure and biochemical comparisons between wild-type and mutated PRPH2 by Protean software. Secondary structure was predicted using Garneier-Robson, Chou-Fasman and Delèage-Roux methods. Green, yellow, blue and orange regions represent alpha helix, beta-sheet, beta turn and coiled coil, respectively. Flexible regions, disorder index, stability-instability index, hydrophilic index and surface probability are highlighted with vermilion, blue and purple, respectively. Differences in mutated prediction are evidenced by red arrows.

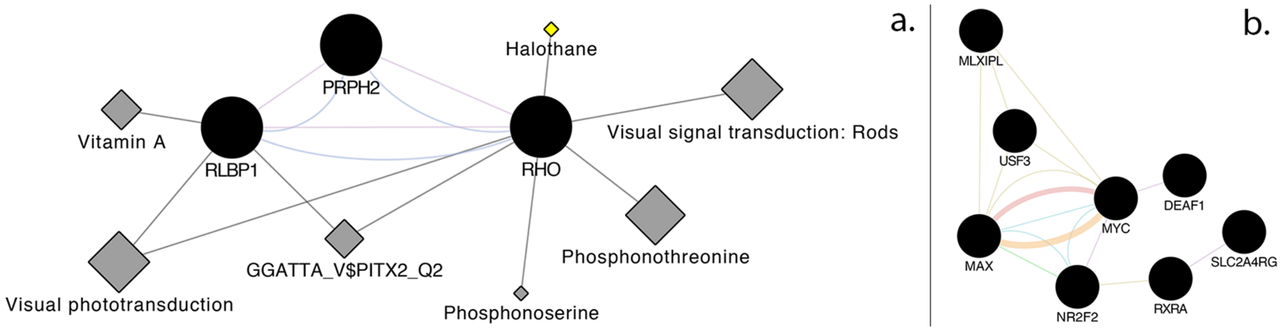

Figure S5. Cytoscape pathway analyses of three analyzed genes and transcription factors with probably altered binding sites. Pathway analysis highlights that PRPH2, RHO and RLBP1 are co-expressed and co-localized in the same tissues (a), probably sharing several common pathways. Furthermore, predictive analysis of proband's RHO and RLBP1 mutated promoters revealed the possible loss of several transcription factor (TF) binding sites. Such TFs probably influence each other, as evidenced by a strong network involving all them (b). Edge colors: genetic interaction (green), co-expression (light purple), shared protein domains (golden yellow), physical interaction (antique pink), predicted (orange), pathway (light blue) and common function (grey).

Table S1. Clash analysis of PRPH2 mutated 3D predicted structure.

SNP ID	*aa Change*	*Adjacent Zone (aa)*	*N° CLASH (WildType)*	*N° CLASH (Mutated)*	*CLASHING aa*
rs390659	Glu304Gln	285, 288, 298, 300 - 307	0	1	Trp306
rs425876	Lys310Arg	308 - 315	0	1	Leu308
rs434102	Gly338Asp	333 - 346	0	30	333 - 336 (Glu, Ala, Glu, Gly)
